# A Pilot Single-Blinded, Randomized, Controlled Trial Comparing BNT162b2 vs. JNJ-78436735 Vaccine as the Third Dose After Two Doses of BNT162b2 Vaccine in Solid Organ Transplant Recipients

**DOI:** 10.3389/ti.2023.10938

**Published:** 2023-04-05

**Authors:** Yoichiro Natori, Eric Martin, Adela Mattiazzi, Leopoldo Arosemena, Mariella Ortigosa-Goggins, Sivan Shobana, David Roth, Warren Lee Kupin, George William Burke, Gaetano Ciancio, Mahmoud Morsi, Anita Phancao, Mrudula R. Munagala, Hoda Butrous, Suresh Manickavel, Neeraj Sinha, Katherine Sota, Suresh Pallikkuth, Julia Bini, Jacques Simkins, Shweta Anjan, Rodrigo M. Vianna, Giselle Guerra

**Affiliations:** ^1^ Miami Transplant Institute, Jackson Health System, Miami, FL, United States; ^2^ Division of Infectious Disease, Department of Medicine, Miller School of Medicine Miami, University of Miami, Miami, FL, United States; ^3^ Division of Hepatology, Department of Medicine, Miller School of Medicine Miami, University of Miami, Miami, FL, United States; ^4^ Division of Nephrology, Department of Medicine, Miller School of Medicine Miami, University of Miami, Miami, FL, United States; ^5^ Department of Surgery, Miller School of Medicine Miami, University of Miami, Miami, FL, United States; ^6^ Division of Cardiology, Department of Medicine, Miller School of Medicine Miami, University of Miami, Miami, FL, United States; ^7^ Division of Pulmonology, Department of Medicine, Miller School of Medicine Miami, University of Miami, Miami, FL, United States; ^8^ Division of Microbiology, Department of Medicine, Miller School of Medicine Miami, University of Miami, Miami, FL, United States

**Keywords:** COVID-19, solid organ transplant, vaccine, booster, randomized controlled trial

## Abstract

Solid Organ Transplant (SOT) recipients are at significant higher risk for COVID-19 and due to immunosuppressive medication, the immunogenicity after vaccination is suboptimal. In the previous studies, booster method showed significant benefit in this population. In the current study, we compared using a mix-and-match method vs. same vaccine as a third dose in SOT recipients. This was a patient-blinded, single center, randomized controlled trial comparing BNT162b2 vs. JNJ-78436735 vaccine as the third dose after two doses of BNT162b2 vaccine. We included adult SOT recipients with functional graft who had received two doses of BNT162b2 vaccine. Participants were randomly assigned to receive either BNT162b2 or JNJ-78436735 in one-to-one ratio. Primary outcome was SARS-CoV-2 IgG positivity at 1 month after the third dose. Sixty SOT recipients, including 36 kidney, 12 liver, 2 lung, 3 heart, and 5 combined transplants, were enrolled, and 57 recipients were analyzed per protocol. There were no statistically significant differences between the two vaccine protocols for IgG positivity (83.3% vs. 85.2% for BNT162b2 and JNJ-78436735, respectively, *p* = 0.85, Odds Ratio 0.95, 95% Confidence Interval 0.23–4.00). Comparison of the geometric mean titer demonstrated a higher trend with BNT162b2 (*p* = 0.09). In this pilot randomized controlled trial comparing mix and match method vs. uniform vaccination in SOT recipients, both vaccines were safely used. Since this was a small sample sized study, there was no statistically significant difference in immunogenicity; though, the mix and match method showed relatively lower geometric mean titer, as compared to uniform vaccine. Further studies need to be conducted to determine duration of this immunogenicity.

**Clinical Trial Registration**: https://clinicaltrials.gov/ct2/show/NCT05047640?term=20210641&draw=2&rank=1, identifier 20210641.

## Graphical Abstract

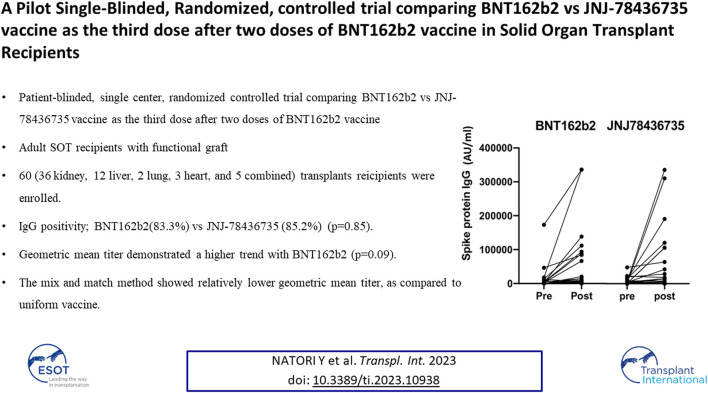

## Introduction

Severe acute respiratory syndrome coronavirus 2 (SARS-CoV-2), known as the etiology behind the coronavirus disease 2019 (COVID-19) worldwide pandemic, has resulted in significant mortality rates worldwide. Solid organ transplant (SOT) recipients, not unexpectantly, are more likely to experience poor outcomes after SARS-CoV-2 infection including higher hospital admission rates and increase mortality (1). In this context, there is an urgent need to provide robust protection in this vulnerable population in addition to standard preventive strategies including wearing mask and hand hygiene.

Other than the natural immunological response against infections, vaccination and monoclonal antibody therapy are the other pathways available to augment the immune systems response to this infection. The United States Food and Drug Administration provided emergency use authorization for ticagevimab/cilgavimab as primary prophylaxis in high-risk patients such as immunocompromised recipients including SOT recipients (2). However, as different variants of concern including Omicron have emerged, the efficacy of some of the monoclonal antibody product has been challenged (3, 4). Thus, the importance of vaccination in this population continues to be a foundation of an effective preventive strategy.

Although the high efficacy of COVID-19 vaccines is well documented in the general population (5), the immunogenicity and efficacy of SARS-CoV-2 vaccination is suboptimal in SOT recipients, something that has been seen in with other vaccines (6). There have been several attempts to improve vaccine efficacy and/or immunogenicity in this vulnerable population, especially with boosted doses. A randomized controlled trial comparing placebo vs. other mRNA vaccine as a third dose study demonstrated significant benefit (7). Furthermore, while this study was being conducted, the addition of a fourth dose has shown to have been beneficial (8), leading to the recommendation of a second booster in the immunosuppressed population. Even with the boosted dose strategy, reports of breakthrough infection in SOT recipients with COVID-19 exist (9).

We hypothesized that the mix and match method, i.e., using the different type of vaccine as a booster, would provide higher immunogenicity in SOT recipients. However, there are two studies comparing the mix and match method vs. uniform method in SOT recipients: one multicenter prospective, non-randomized, study and one randomized controlled trial (10, 11). The former vaccine series of Schwaighofer et.al. cohort differed from our study by utilizing various vaccines such as mRNA-1273 and BNT162b2 prior to administration of the third dose of AD26COVS1(10). Chiang et.al. conducted a prospective observation study, which cannot avoid selection bias (11). To study this concept more carefully, we conducted a single center randomized controlled trial comparing BNT162b2 (mRNA vaccine) vs. JNJ-78436735 (viral vector) as a third dose after completion of two doses of BNT162b2 vaccine in SOT recipients.

## Patients and Methods

### Patient Selection and Study Design

This was a patient-blinded, superiority, randomized controlled trial, conducted at the Miami Transplant Institute, Jackson Health System, Miami, Florida, USA. The Miami Transplant Institute is one of the biggest SOT centers in North America, providing comprehensive care to all SOT recipients.

We included SOT recipients with a functional graft, whose age was 18 years and older at the time of enrollment. Inclusion for enrollment consisted of recipients with a minimum of 1 month post-transplant and having received two doses of BNT162b2 vaccine. Of note, the prior vaccines could have been administered any time pre or post transplantation. The third dose should have been given at least 28 days from the second dose of BNT162b2 vaccination and at least 1-month post-transplant. Exclusion criteria included any significant side effects due to previous SARS-CoV-2 vaccination, people unable to consent, receipt of more than or equal to three doses of SARS-CoV-2 vaccination, pregnancy and patients who previously received monoclonal antibody treatment that are specifically directed against the spike protein for SARS-CoV-2 such as Bamlanivimab plus Etesevimab, Casirivimab plus Imdevimab, and Sotrovimab at any time prior to the trial. Of note, at the time of enrollment, Ticagevimab/Cilgavimab was not available in USA.

This study was approved by local research ethics board and was given NCT05047640.

### Blinding, Unblinding, Randomization and Follow up

After obtaining written informed consent, adult SOT recipients were randomized in one to one ratio to receive either BNT162b2 vs. JNJ-78436735. BNT16b2 uses nucleoside-modified mRNA encoding the viral spike glycoprotein for SARS-CoV-2 as an ingredient. On the other hand, JNJ-78436735 uses recombinant, replication-incompetent Adenovirus 26 vector, encoding a stabilized variant of SARS-CoV-2 spike protein, as an ingredient. A randomization schedule was created electronically and simple randomization was performed. The participants’ blood specimens were collected to analyze anti-spike protein SARS-CoV-2 IgG. The patients were contacted by phone at day 3 and 7 post vaccination to monitor for adverse events. Follow-up blood test was planned between 21 and 35 days after the third dose of the vaccine to measure anti-spike protein SARS-CoV-2 IgG. We measured IgG titer to the SARS-CoV-2 spike protein receptor binding domain using enzyme-linked immunosorbent assay as described elsewhere (12). Briefly, the SARS-CoV-2 enzyme-linked immunosorbent assays were performed following a 2-step enzyme-linked immunosorbent assay protocol and results were interpreted in accordance with the manufacturer’s cutoff calculations. Anti-spike protein SARS-CoV-2 IgG was reported as receptor binding domain (RBD) (13). At that time, we also questioned the adverse events. The vaccine given at the time of enrollment was unblinded at the time of follow up blood test to the participant. However, if an emergency ensued, the vaccine could be unblinded immediately for the patient and caring team.

Of note, this study was not observer blinded. However, the laboratory members were not notified of the randomization results.

### Statistical Analysis and Sample Size Calculation

The primary outcome of the study was anti-spike protein SARS-CoV-2 IgG positivity after 28 (21–35) days of the booster dose with either vaccine. Secondary outcomes included side effect, graft rejection, and SARS-CoV-2 infection. The follow-up period of the current study was 28 (21–35) days, up to the follow-up blood collection. We set alfa of 0.05 and beta of 0.2. For pre-specified outcome analysis, based on our hypothesis, we compared IgG positivity between two vaccines. As an ancillary analysis, we tried to identify the risk factors to develop or not to develop IgG positivity in this cohort. We assumed the anti-spike protein SARS-CoV-2 IgG positivity in JNJ-78436735 as 80% and BNT162b2 as 60% (7). The number of subjects required for this analysis was 93 per each arm, or a total of 186. We assumed 5%–10% of patients would be lost to follow-up. Therefore, we planned to enroll 200 patients in total, to achieve statistical significance per protocol sample.

Demographics were analyzed using descriptive statistics. Pre- and post-vaccination anti-spike protein SARS-CoV-2 IgG titers were compared using Wilcoxon rank-sum test. Univariate analyses were performed to determine significant factors affecting seroconversion using chi-squared or Fisher’s exact test for categorical variables and Mann–Whitney U for continuous variables. For multivariate analysis, we planned to construct a model using variables whose *p*-value were less than 0.2 on univariate analysis. Multivariate analysis was performed using logistic regression with stepwise backward elimination. Statistical significance was defined as a *p*-value of less than 0.05. Statistical analysis was performed using SPSS version 26.0 (Chicago) and GraphPad Prism version 8.0 (La Jolla, CA, USA).

## Results

### Patient Population

**FIGURE 1:**
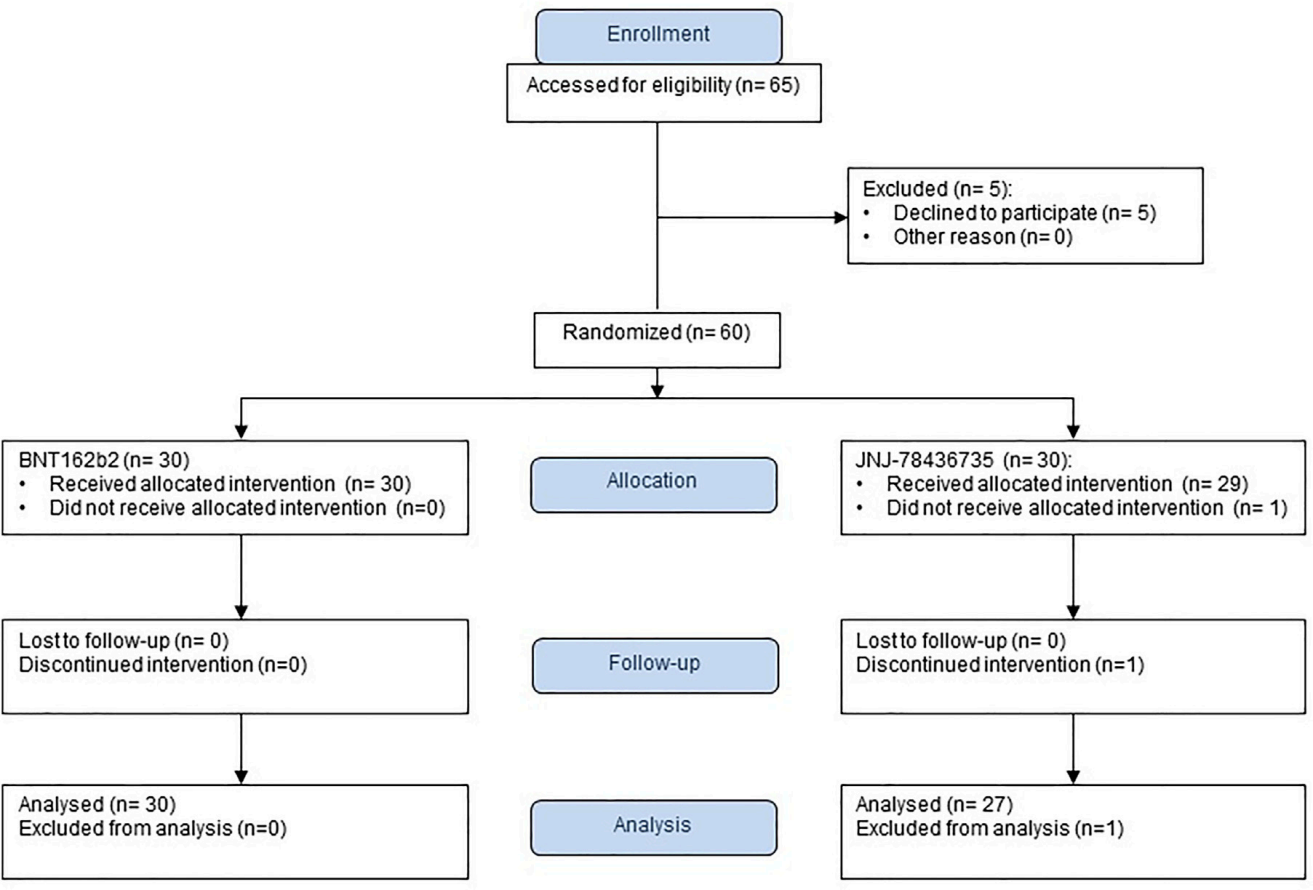
Study flow chart.

From September to December 2021, we enrolled 60 SOT recipients and 59 of them received a study vaccine as one patient withdrew after obtaining the consent, prior to vaccination (30 BNT162b2, 29 JNJ-78436735) (Figure 1). We could not enroll the number of recipients because the majority of them had already received the third dose. The termination was not due to the interim analysis. After enrollment, one patient declared that he had received monoclonal antibody, (resulting in the withdrawal of that participant (30 BNT162b2, 28 JNJ-78436735). Finally, we enrolled 36 kidney, 12 liver, 2 lung, 3 heart, and 5 combined. Baseline characteristics of 58 enrolled patients were shown in Table 1. The overall median time from transplant and the second dose of BNT162b2 to study vaccination was 10.7 [IQR] (4.7–38.4) and 7.8 (IQR 6.6–8.3) months, respectively. Of note, 20/58 (34.5%) of the recipients received the prior two doses prior to transplant. Only ethnicity was different between both groups (*p* = 0.02). Other demographic characteristics including type of transplant, presence of recent rejection, and immunosuppression at the time of vaccination were well balanced in the two groups.

**TABLE 1:** Patient characteristics at enrollment.

Characteristic	All (n = 58)	BNT162b2 (n = 30)	JNJ-78436735 (n = 28)
Age, median (range)	57.5 (26–79)	59.5 (27–76)	54.5 (26–79)
Male sex (%)	38 (65.5)	21 (70)	17 (60.7)
Time from transplantation to vaccination (months), median (interquartile range)	11.5 (3–27)	10.7 (4.7–38.4)	12.5 (2.8–25.7)
Within 1 year of transplantation (%)	30 (51.7)	16 (53.3)	14 (50.0)
History of documented COVID-19(%)	7 (12.1)	4 (13.3)	3 (10.7)
Receipt of Anti-thymocyte globulin^a^ (%)	17 (29.3)	8 (26.6)	9 (32.1)
Recent Rejection (%)	14 (24.1)	7 (23.3)	7 (25.0)
Type of transplant (%)			
Kidney	36 (62.0)	19 (63.3)	17 (60.7)
Liver	12 (20.7)	3 (10)	9 (32.1)
Lung	2 (3.4)	2 (6.7)	0 (0)
Heart	3 (5.2)	3 (10.0)	0 (0)
Combined	5 (8.6)	3 (10.0)	2 (7.1)
Immunosuppression			
Prednisone (%)	25 (43.1)	14 (46.7)	11 (39.2)
Prednisone dose, mg/day, median (range)	5 (2.5–80)	5 (2.5–80)	7.5 (4–40)
Tacrolimus (%)	52 (89.7)	26 (86.7)	26 (92.9)
Mycophenolate mofetil/mycophenolate sodium (%)	46 (79.3)	25 (83.3)	21 (75.0)

^a^ Within 6 months prior to the third dose of vaccination.

### Vaccine Immunogenicity

Of the 58 patients who were successfully vaccinated, one recipient that had received JNJ-78436735 was not included for the immunogenicity analysis due to acquiring SARS-CoV-2 infection prior to the second blood draw (Figure 1). The remainder of the recipients completed pre- and post-vaccination sera collection. Therefore, 57 patients were available for the immunogenicity analysis (30 BNT162b2, 27 JNJ-78436735) (Figure 1).

Post vaccination immunogenicity rates, which is the primary outcome, for BNT162b2 and JNJ-78436735 were 83.3% and 85.2% respectively (*p* = 0.85, Odds Ratio 0.95, 95% Confidence Interval 0.23–4.00).

The baseline anti-spike protein SARS-CoV-2 IgG positive rate was 36.9% among all cohort and there was no statistically significant difference between BNT162b2 and JNJ-78436735. Median quantitative SARS-CoV-2 IgG titers at the time of enrollment for BNT162b2 and JNJ-78436735 were 719 (range 11–173057) AU/mL and 2385 (range 101–48296) AU/mL, respectively.

**FIGURE 2:**
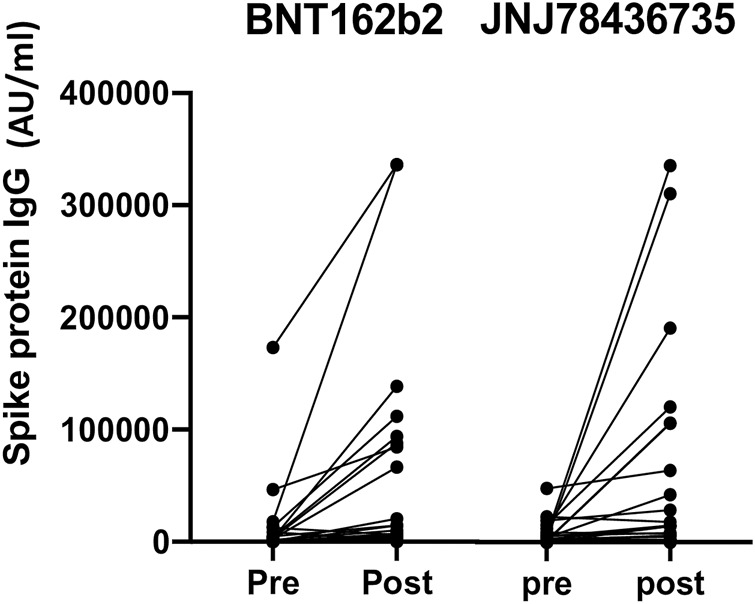
Quantitative anti-spike protein IgG titer pre and post third dose of either BNT162b2 or JNJ78436735. Each dot represents each participant’s IgG titer at pre or 1 month post third dose of vaccination.

Quantitative anti-spike protein SARS-CoV-2 IgG increased significantly post third dose vaccination compared to baseline (*p* < 0.001) in entire cohort (Figure 2).

Median geometric mean titer (GMT), analyzed as the absolute fold-increase of titer from pre- to post- third dose of the vaccination, for BNT162b2 and JNJ-78436735 was 9.51 (range 0.18–284.54) and 1.64 (range 0.24–170.2), respectively and there was a trend towards BNT162b2 showing higher response (*p* = 0.09).

When proceeding to analyze factors affecting vaccine IgG positivity after vaccination, we found in the univariate analysis that none of the variables could be identified as risk factors since all *p* values were greater than 0.2. Of note, we have analyzed age, gender, race, transplanted organ, duration between transplant and vaccination, recent rejection, usage of immunosuppressive medication including prednisone, tacrolimus, mycophenolate and anti-thymocyte globulin. Hence, we did not conduct multivariate analysis.

### Vaccine Adverse Events

Vaccine-related adverse events were assessed in the 58 patients who received study vaccine (Figure 1). During follow-up, there were no statistically significant differences for local and systemic side effects in both groups (Table 2). The most common adverse event reported was localized injection site pain (14/58, 24.1%), which were seen within 7days after the vaccination. None of the 58 patients were diagnosed with new onset of rejection during the follow up. Mild SARS-CoV-2 infection was diagnosed in one patient at 31 days after JNJ-78436735 vaccination.

**TABLE 2:** Adverse Events after vaccination.

	BNT162b2 (n = 30)	JNJ- 78436735 (n = 28)
Local		
Arm Pain	8 (26.7)	6 (21.4)
Erythema	1 (3.3)	0 (0)
Any local reaction	9 (30.0)	6 (21.4)
Systemic		
Headache	3 (10.0)	2 (7.1)
Fatigue	5 (16.7)	2 (7.1)
Muscle aches/Joint pain	0 (0)	0 (0)
Gastrointestinal symptoms	0 (0)	0 (0)
Fever/Chills	1 (3)	1 (3.5)
Thrombosis	0 (0)	0 (0)
Any systemic reaction	7 (23)	5 (17)

## Discussion

This was a randomized controlled trial comparing BNT162b2 vs. JNJ-78436735 as a third dose after completion of two doses of BNT162b2 in SOT recipients. Similar to previous randomized controlled trial (10) and non-randomized large observational study (11), these two vaccines were safely used in this population with similar immunogenicity as shown. Due to small sample size, not only the primary outcomes but also the secondary analysis, including risk factor analysis, may be inconclusive. However, although not statistically significant, we observed slightly higher immunogenicity following vaccination with mRNA vaccine.

At the time of our trial, there were two studies assessing the immunogenicity of mixing method in SOT recipients. One single center randomized controlled trial, conducted by Schwaighofer et al. (10), compared mRNA vaccine (either BNT162b2 or mRNA-1273) vs. Ad26COVS1 in 197 kidney transplant recipients with negative responses after two doses of mRNA vaccine. The positive antibody responses against SARS-CoV-2 spike protein after mRNA vaccine vs. Ad26COVS1 were 35% and 42%, respectively, not statistically significant. The other trial by Chiang et. al. concluded that mixing method did provide higher rate of seroconversion at 3- and 6-months post third dose vaccination in contrast to our study where GMT was higher in uniform method group. As a hypothesis, there might be an additive synergistic effect accompanying the administration of the same vaccine in contrast to the results seen using the mixing method. Of note, currently, JNJ-78436735 COVID-19 vaccine is authorized for adults only in certain limited situations due to risk of thrombosis with thrombocytopenia syndrome.

There are several limitations in this current study. Sample size was never achieved due to the challenges of persuading patients to possibly receive different vaccines based on randomization. Of note, the majority of our recipients had received the third dose at the time of enrollment. In addition, the prior vaccines could have been administered pre- or post transplantation; 34% of participants were vaccinated before transplant. Thus, we cannot conclude whether results are comparable between those vaccinated pre- and post-transplantation. In this study, we are limited to the use of surrogate marker, not the incidence itself. We included not only seronegative but also seropositive recipient at the time of the third dose vaccination in order to most accurately reflect our current population. We tried to address this limitation by calculating GMT. Lastly, our follow up consisted of 1 month duration making challenging to capture late occurring adverse events, along with concluding that IgG positivity 30 days post third vaccine dose properly reflect long term immunogenicity in transplant recipients. This warrants longer follow up for future studies.

In conclusion, we conducted a patient-blinded, randomized controlled trial comparing BNT162b2 vs. JNJ-78436735 vaccine for the third dose after two doses of BNT162b2 COVID-19 vaccines in SOT recipients. We found similar immunogenicity using both vaccination strategies. Even though the primary outcome was not achieved due to small sample size being underpowered, larger studies will need to be performed to draw conclusion. Further investigation is needed to understand the optimal method of COVID-19 vaccination in this vulnerable group of patients. Also, further studies need to be conducted to determine duration of this immunogenicity.

## Data Availability

The original contributions presented in the study are included in the article/supplementary material, further inquiries can be directed to the corresponding author.

## Abbreviations

COVID-19, Coronavirus disease 2019; GMT, Geometric mean titer; RBD, Receptor binding domain; SARS-CoV-2, Severe acute respiratory syndrome coronavirus 2; SOT, Solid Organ Transplant.
